# Common Traits Spark the Mitophagy/Xenophagy Interplay

**DOI:** 10.3389/fphys.2018.01172

**Published:** 2018-09-20

**Authors:** Aarti Singh, Sharon L. Kendall, Michelangelo Campanella

**Affiliations:** ^1^Department of Comparative Biomedical Sciences, Royal Veterinary College, London, United Kingdom; ^2^Department of Pathology and Pathogen Biology, Royal Veterinary College, Hertfordshire, United Kingdom; ^3^UCL Consortium for Mitochondrial Research, London, United Kingdom

**Keywords:** xenophagy, mitophagy, TBK1, TSPO, mitochondria, bacteria

## Abstract

Selective autophagy contributes to the wellbeing of eukaryotic cells by recycling cellular components, disposing damaged organelles, and removing pathogens, amongst others. Both the *quality control* process of selective mitochondrial autophagy (Mitophagy) and the *defensive process* of intracellular pathogen-engulfment (Xenophagy) are facilitated via protein assemblies which have shared molecules, a prime example being the Tank-Binding Kinase 1 (TBK1). TBK1 plays a central role in the immunity response driven by Xenophagy and was recently shown to be an amplifying mechanism in Mitophagy, bring to attention the potential cross talk between the two processes. Here we draw parallels between Xenophagy and Mitophagy, speculating on the inhibitory mechanisms of specific proteins (e.g., the 18 kDa protein TSPO), how the preferential sequestering toward one of the two pathways may undermine the other, and in this way impair cellular response to pathogens and cellular immunity. We believe that an in depth understanding of the commonalities may present an opportunity to design novel therapeutic strategies targeted at both the autonomous and non-autonomous processes of selective autophagy.

## Introduction

Running comparative investigations on species-specific processes allows the comprehension of the underlying biological phenomena; thus, better framing their general value and devising accurate strategies of intervention. Studies on Xenophagy and Mitophagy are steadily bringing to light shared elements between these two evolutionary divergent selective types of autophagy detailing their molecular biology and inspiring novel approaches of exploitation.

Autophagy patrols the intracellular environment and can do so selectively by targeting either mitochondria (mitophagy) (Lemasters, 2005), protein aggregates (aggrephagy) (Lamark and Johansen, 2012), lipids (lipophagy) (Weidberg et al., 2009) or pathogens (xenophagy) (Levine, 2005) with new selective autophagy mechanisms being discovered continuously. These means of cellular quality control rely on molecular mechanism, which may be common between them and therefore account for a subtle interplay to which little attention has been devoted.

The recent advancements on the molecular function of Tank-binding kinase 1 (TBK1) unveiled a role in mitophagy thus complementing the established one in Xenophagy (Thurston et al., 2009; Wild et al., 2011; Pilli et al., 2012) This has provided us with an opportunity to discuss values and dangers of a similar molecular co-sharing besides posing novel questions on core regulatory aspects of mammalian cells homeostasis in health and disease.

In this short contribution, we shall snapshot the fundamentals of Mitophagy and Xenophagy with the aim of highlighting the relevance of common elements and in this way pave a path forward to learn how to enhance or inhibit their unfolding for potential therapeutic benefit.

## How Selective Autophagy Protects Against Extra- and Intracellular Toxic Elements

Autophagy is the conserved and genetically programmed homeostatic process which traps and degrades intracellular components that are no longer necessary or have become dysfunctional or damaged (Mizushima and Klionsky, 2007; Yang and Klionsky, 2010a). It targets damaged or excessive organelles by engulfing them into a double-membraned autophagosome which ultimately fuses with lysosomes for degradation (Levine and Klionsky, 2004; Mizushima, 2007; Glick et al., 2010). Examples of autophagy regulators include: autophagy-related genes (ATGs) (Itakura and Mizushima, 2010), mechanistic target of rapamycin complexes (mTORC1) (Wong et al., 2015), beclin-1 (mammalian ortholog of Atg6) (Kang et al., 2011), unc-51 like autophagy activating kinase 1 (ULK1) (Russell et al., 2013) and Microtubule-associated proteins 1A/1B light chain 3B (LC3) (Glick et al., 2010; Yang and Klionsky, 2010b).

The main process of macroautophagy, considered to be the main form of autophagy, a double-membraned phagophore is formed around ubiquitinated proteins or organelles, which matures into an autophagosome that ultimately fuses with a lysosome (Feng et al., 2014). Whereas, during the process of microautophagy the substrates are directed into the lysosome through invagination resulting in their degradation (Li et al., 2012). The chaperone Mediated Autophagy (CMA) occurs instead through the recognition of a specific motif to which the chaperone complex binds and forms a substrate/chaperone complex that fuses with the lysosome upon recognition of the CMA receptor (Kaushik et al., 2011; Kaushik and Cuervo, 2012).

**FIGURE 1:**
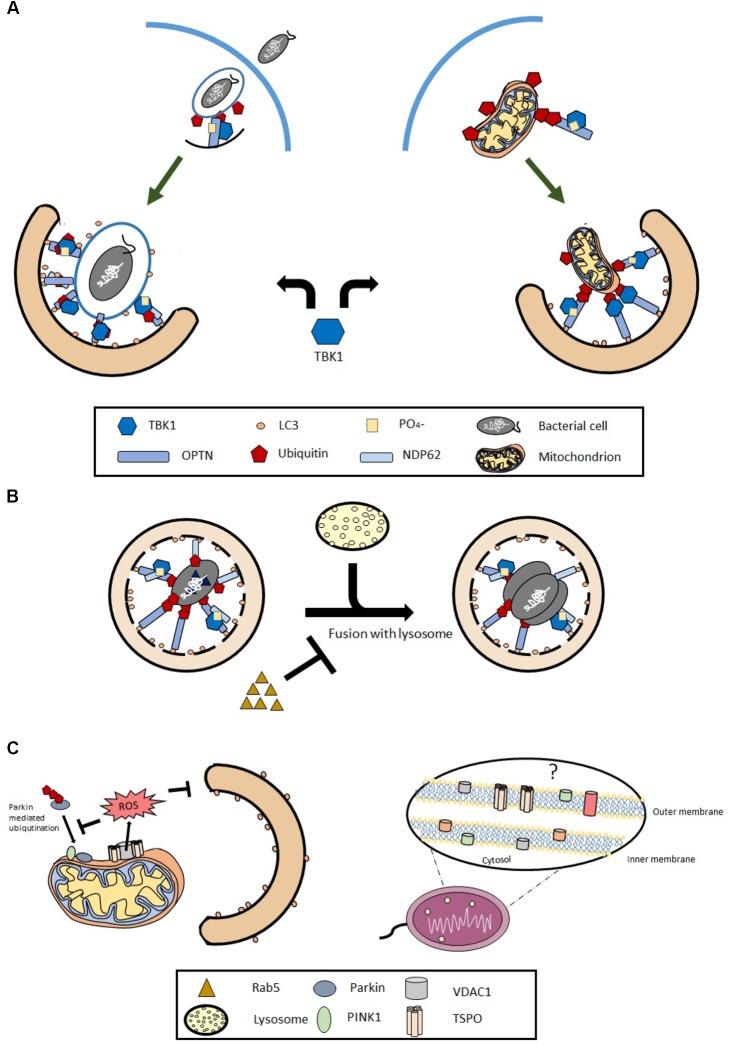
Tank-binding kinase 1 (TBK1) as a common functional element between Xenophagy and Mitophagy. Panel **(A)** depicts pivotal steps in the two processes of selective autophagy against pathogens and mitochondria in which TBK1 plays an equally important role. Panel **(B)** highlights instead that the similarity may embrace also inhibitory mechanisms among which the TSPO pathway is proposed **(C)**.

Core elements of the autophagy machinery are retained in the selective versions of the process of which the most extensively characterized versions are: (i) Mitophagy which consists of the degradation of dysfunctional or damaged mitochondria (Youle and Narendra, 2011; Jin and Youle, 2012) and (ii) Xenophagy which is instead the removal of invading pathogens such as bacteria and viruses (Knodler and Celli, 2011; Mao and Klionsky, 2017) (**Figure 1**). Mitophagy and Xenophagy are finely tuned processes, which share key steps such as the ubiquitination of the unwanted elements prior their disposal via the autophagy-lysosomal pathway (Alomairi et al., 2015). Both processes depend on three key steps: flagging the problem (ubiquitination), fusing with degradative machinery (autophagosome and lysosomal fusion) and breakdown (acidic and enzymatic degradation). These common elements may represent co-regulatory framework.

The innate immune system is the frontline defense against pathogens, which also acts as a bridge for the adaptive immune response to further control and prevent the invasion (Iwasaki and Medzhitov, 2015). Innate immunity functions through a multitude of signaling pathways, which are conserved across species and grant organisms the fundamental ability to make a distinction between *self* and *non-self* (Mogensen, 2009) with autophagy playing part in this (Deretic, 2011). Xenophagy is the activation of a selective breakdown specifically in the context of invading microbial organisms by contributing the prominent processes of phagocytosis and recognition (**Figure 1**). Xenophagy is distinct from the biological process of phagocytosis as the former acts as a specialized protective mechanism for cells which have already been targeted and breached by pathogens (Flannagan et al., 2009) while phagocytosis is not specific to pathogens alone, and is often utilized to engulf other cells or debris as well.

When pathogens undergo recognition through pattern recognition receptors (PRRs) (Levine and Klionsky, 2004) whereby PRRs identify the pathogen associated molecular patterns (PAMPs). This then initiates the immune signaling preceding the internalization of the pathogen and the activation of the autophagy machinery resulting in entrapment in autophagosomes once within the cytosol and subsequent autolysosomal degradation (Delgado et al., 2009; Oh and Lee, 2014). This is particularly relevant in mammalian cells, which adopt cytosolic or cell surface bound PRRs [such as Toll-like receptors (TLRs) or NOD-like receptors (NLRs)] to detect invading pathogens and signal the upregulation of targeted Autophagy via Xenophagy (Sanjuan et al., 2007).

Xenophagy relies on components of the immunity pathways such as Stimulator of interferon genes (STING) and galectin-8 (Thurston et al., 2012; Watson et al., 2012) which act as cytosolic sensors of the pathogen and recruit downstream effectors (Crotzer and Blum, 2010). Studies have now shown that when the MHC class I protein surface expression is diminished (Li et al., 2010; Oh and Lee, 2014) a reduction in the levels of Xenophagy occurs thus implying that a response could not be successful without selective autophagy embedded and functional therein.

Xenophagy and Mitophagy are both mediating selective disposal of unfit elements and therefore considered to be part of an immune-like response key to maintain cellular homeostasis (**Figure 1**).

Mitochondria are pivotal to cellular function as producers of the majority of cellular adenosine triphosphate (ATP), intracellular signaling decoders and docking base for cyclic adenosine monophosphate (cAMP) effectors (Tarasov et al., 2012; Finkel et al., 2015; Zhang et al., 2016). Mitochondria are not originally part of the ancestral cell as they are likely of bacterial origin which make of them “*hosted elements*” despite the successful co-habitation (Embley and Martin, 2006; Gray, 2012). There are several theories describing how mitochondria ended up in mammalian cells; the most prominent of which is the endosymbiosis (Martin et al., 2001; Archibald, 2015), whereby the mitochondrion was originally an extracellular organism [likely α-proteobacterial (Andersson et al., 1998)] capable of oxidative phosphorylation and therefore engulfed in eukaryotic cell to improve the energetic capacity (Taanman, 1999; Thiergart et al., 2012; Martin et al., 2015). This evolved into a successful symbiotic relationship, which crossed evolution. Mitophagy may have evolved as a response to this, thus acting as a controller for these foreign organelles. Mitophagy recognizes and clears the cell of damaged mitochondria preventing the accumulation of dysfunctional mitochondria harmful to the intracellular environment. Even though it exploits the same upstream initiators to general autophagy, the overall mitophagic response is regulated by process-specific proteins to distinguish *damaged self* from *integer self* within the mitochondrial network (Ding and Yin, 2012).

Given their origin, the adaptation of the mitochondrion to the early ancestral eukaryotic cell would require a unique subset of proteins to sense and regulate the organelle. Examples include the PTEN-induced kinase 1 (PINK1) which is capable of recognizing dysfunctional mitochondria (Jin and Youle, 2012). It is expressed at very low levels in healthy mitochondria due to successful cleaving of the protein into smaller products after its import into the inner mitochondrial membrane. If a mitochondrion is damaged, however, full-length PINK1 will accumulate on the outer membrane. This leads to the recruitment of the E3 ubiquitin ligase Parkin which ubiquinates the mitochondria, tagging them for lysosomal degradation (Jin and Youle, 2013; Kane et al., 2014; Hamacher-Brady and Brady, 2016).

Notably, various conditions exploit this pathway leading to its impairment spanning from metabolic diseases (e.g., Fanconi anemia) to neurodegeneration, all leading to persistent cellular and tissue damage. Dysfunctional mitochondria can lead to cytotoxicity (Nicholls, 2002; Akbar et al., 2016), hyperactivation of the NLRP3 inflammasome (Lopez-Armada et al., 2013) and cell death via uncontrolled release of the Cytochrome *c* (Kubli and Gustafsson, 2012). Mitophagy therefore maintains the balance of multiple cellular signaling pathways, downregulating ROS production and helping to maintain a healthy population of mitochondria in the cell (Lazarou, 2014).

Another common element between Mitophagy and Xenophagy is that may remain functional in absence of ubiquitin. In ubiquitin-independent mitophagy mitochondrial receptors like Nip3-like protein X (Nix) (Koentjoro et al., 2017) and FUNDC1 (Liu et al., 2012) interact directly with LC3 (and hence with the autophagosome) leading to lysosomal degradation. Ubiquitin-independent Xenophagy sees galectin-8 capable of recognizing the glycans of the vacuole within which the pathogen resides: this recruits the cargo receptor NDP52 (CALCOCO2) to complete degradation via autophagy (Thurston et al., 2012). In addition, the LC3-associated phagocytosis (LAP), a novel form of non-canonical autophagy, can also be considered an ubiquitin-independent type of Xenophagy hijacking components of the autophagy machinery to aid phagocytosis of extracellular particles and pathogens (Martinez et al., 2015). In LAP LC3 is quickly covering the phagosome for a rapid fusion with lysosome resulting in degradation without pro-inflammatory immune response (Heckmann et al., 2017; Schille et al., 2018).

Whether Mitophagy should be considered equal to Xenophagy in defining the immune response process is debatable. Undeniable though is that operates as an adaptor via mechanisms (memory based) resembling features of immunity exploited against mitochondria (Krysko et al., 2011). Can Mitophagy inform Xenophagy and vice-versa? Are there common functional elements between the two, which could dictate their mutual influence and dictate their efficiency according to the physiopathology of the cell? The recent advances on TBK1 imply this may be highly plausible.

## The Uncovering of Another Common Conduit

The Serine/threonine-protein kinase, TBK1, is known to be required in Xenophagy to maintain structural integrity of the pathogen-containing vacuoles. Studies have convincingly shown that knocking down of TBK1 as well as of NDP52, with which it complexes, results in defective clearance of bacteria allowing their escape into the cytosol (Radtke et al., 2007; Thurston et al., 2009; Pilli et al., 2012). The cargo-associated “eat-me” signals as well as the receptors mediating selective autophagy to bridge cargo and phagosomes have been previously unveiled for both processes as reviewed by Randow and Youle (2014).

In 2016, Dikic and colleagues showed that TBK1 integrates the ubiquitin dependent signaling events in Mitophagy upstream of the process (Heo et al., 2015; Moore and Holzbaur, 2016; Richter et al., 2016). They have convincingly shown that TBK1 phosphorylates the Mitophagy receptor Optineurin (OPTN), on the ubiquitin-binding domain (UBD) and the LC3-binding ones. Via this processing TBK1 control and regulates the degradation of dysfunctional mitochondria to which it is selectively recruited. TBK1 mediates phosphorylation of OPTN on the S473 thus expanding the binding capacity of OPTN to multiple ubiquitin chains necessary for both TBK1 recruitment and OPTN targeting to ubiquitinated mitochondria. Mutated TBK1 instead fails to phosphorylate OPTN and therefore stalls the downstream signaling cascade for the activation of Mitophagy. This process of phosphorylation is also implicated in the Parkin independent and PINK1 mediated Mitophagy, highlighting an important molecular partnership in the regulation of homeostatic Mitophagy. Dikic and colleagues were also able to show that multiple Mitophagy receptors beyond OPTN are targeted by TBK1 such as NDP52 (CALCOCO2), TAX1BP1, and p62 (SQSTM1).

This TBK1-mediated phosphorylation establishes therefore an amplification loop that activates the molecular pathway driving the selective degradation of mitochondria.

In Xenophagy the identification of the Serine/threonine-protein kinase TBK1 was paradigm shifting since it helped to clarify the regulatory and recruitment mechanisms of pathogen ubiquitin regulation. The wealth of subsequent literature better contextualized the significance of TBK1 as amplifying signaling in Xenophagy (Weidberg and Elazar, 2011; Helgason et al., 2013; Yang et al., 2016).

Being now aware that TBK1 is required to amplify the removal of both invading pathogens and damaged mitochondria makes us wonder whether defective Mitophagy may indirectly impact Xenophagy. Explicitly, whether defective Mitophagy may undermine Xenophagy recruiting pools of TBK1 to undertake the process (i) or whether a high degree of Xenophagy could impact the unfolding of Mitophagy (ii). Above all interesting to encrypt would be whether a detectable hierarchy exists between the two processes.

The potential cross talk between these two processes, as well as the mutual hijacking of core molecules here hypothesized, calls for further studies which should begin by considering the inhibitory mechanisms of both these processes which we detail below.

## The Implications for Common Conduits

Pathogens have evolved distinct mechanisms to evade Xenophagy, particularly by avoiding autophagic consumption. There are a variety of methods that pathogens exploit to avoid lysosomal degradation. These include: (i) creating a neutral compartment within cells where the pathogen can replicate and then escape as exploited by Brucella abortus (Case and Samuel, 2016); (ii) Hijacking the pathway and persisting within quiescent membrane reservoirs inside the autophagosome to later re-establish recurrent infections as done by the uropathogenic *Escherichia coli* (UPEC) (Mulvey et al., 2001; Mysorekar and Hultgren, 2006; Lewis et al., 2016); (iii) Mycobacterium Tuberculosis (MT) instead, prevents the maturation of the phagosome into the autolysosome by releasing inhibitory factors of the likes of ESAT-6 and Rab5 (Chandra et al., 2015; López de Armentia et al., 2016; Russell, 2016).

It can therefore be considered that the evolutionary selective pressure exerted by the innate immune response, in the form of Xenophagy, has driven adaptation in pathogens for enhanced virulence.

**FIGURE 2:**
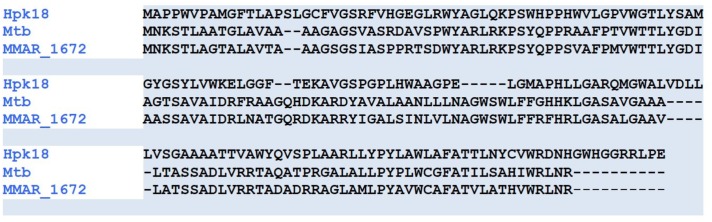
Sequence alignment of mycobacterial TspOs with the human homolog. Sequences were aligned using Clustal Omega and show approximately 30% amino acid identity with the human homolog. Stars indicate identical amino acid residues and dots indicate semi-conserved (similar residues) in all three sequences.

Concomitantly, the subversion of Xenophagy by pathogens could have evolved to form a symbiotic relationship and underpin the successful co-habitation of mitochondria within the hosting cell. This is particularly relevant for a pathogen like MT and UPEC which can lie in a state of dormancy for many years and cause diseases which are increasingly difficult to treat. Based on this, Mitophagy-inhibiting molecules could exploit the same evasion mechanisms as understood with Xenophagy. Hitherto, there is one prominent molecule described as Mitophagy inhibitor: the mitochondrial Translocator Protein (TSPO) (Gatliff et al., 2014) whose role in Xenophagy remains unaddressed (**Figure 1**) in spite of its high degree of conservation between mammalian and bacterial genomes (Li et al., 2016). A bacterial homolog of the mammalian TSPO, Tryptophan-rich sensory protein (TspO) was first identified in the photosynthetic bacterium Rhodobacter Sphaeroides (RS), where it is thought to be enrolled in the biosynthetic pathway for photosynthetic pigments acting as a negative regulator of photosynthesis genes in response to light and oxygen availability (Yeliseev and Kaplan, 1999). Since its initial discovery in RS, *Tspo* homologs have been found in a wide range of bacterial taxonomic groups (Chapalain et al., 2009) and human pathogens such as Bacillus anthracis, Legionella pneumophila, Staphylococcus haemolyticus, and Clostridium perfringens. Expression of tspO in Pseudomonas fluorescens increases adhesion and decreases apoptosis (Chapalain et al., 2009) (**Figure 2**). These observations suggest a role for bacterial TspOs in virulence, particularly when this is delivered intracellularly. It is therefore arguable that bacterial tspOs might represent a conduit to: a) give further insight on the Xenophagy evasion mechanisms exploited by intracellular parasites and b) Enlighten on the crosstalk with Mitophagy (**Figure 1**).

In this regard, what if mammalian cells overexpressing TSPO (and subsequently bearing impairment in the cellular mitophagic response) undermine xenophagy, enabling the establishment of bacterial infections? We are tempted to speculate that if Mitophagy requires greater commitment by the machinery dedicated to the process (such as in the cases in which TSPO is overexpressed) this is likely to de-potentiate Xenophagy. Intriguingly, we know nothing of the ability of TBK1 to retain its amplificatory role in both Mitophagy and Xenophagy in presence of inhibitory elements such as TSPO thus posing the question whether unfolding of the processes are preserved during pathological conditions, as it is known that TSPO is generally overexpressed in these (Liu et al., 2014; Roncaroli et al., 2016).

The possibility for which defective Mitophagy could undermine the efficiency of Xenophagy has never been properly contemplated nor considered in depth, in spite of some evidences available in the literature. The *Streptomyces antibioticus*, for example, is capable of producing antimycin, an inhibitor of the respiratory chain complex III (Rehacek et al., 1968) also used in combination to trigger mitophagy. In line with this Francione et al., 2009 reported that patients suffering from mitochondrial diseases show an increased susceptibility for infection by legionella supporting that pathogens could well exploit Mitophagy enhancing factors to repress Xenophagy.

**FIGURE 3:**
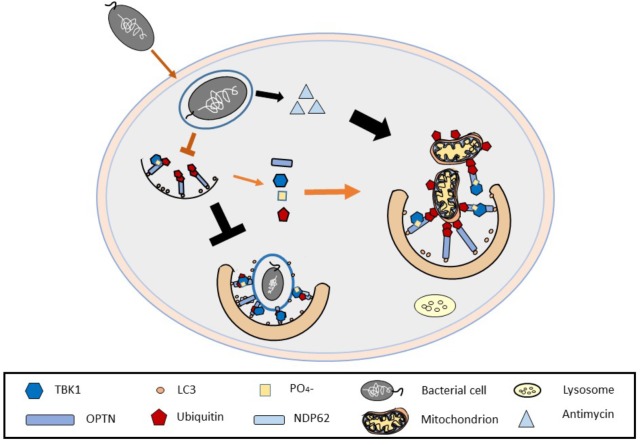
Undermining mitochondria to establish bacterial infections. The figure depicts the ability of certain bacterial species to release mitochondria impairing toxins (i.e., antimycin to impair mitochondria) which upregulate mitophagy, perhaps as a way to hijack the common molecules used in both autophagy processes and as such allow the bacteria to propagate within the host cell.

The above evidence, as well as the mechanistic advancements on TBK1 and the parallel characterization of anti-mitophagic stress response elements such as TSPO (Gatliff et al., 2014) make us speculate that if amplificatory mechanisms are required for Mitophagy completion, this is likely impaired and therefore the recruitment of the shared elements, may deprive Xenophagy of core elements for its proper unfolding allowing the spread of invading pathogens (Gatliff and Campanella, 2016; Kimmey and Stallings, 2016) (**Figure 3**).

We conclude that the unveiling of common elements may represent a viable approach to succeed in manipulating cellular fate and with it the ability to combat diseases and disorders caused by deficient or abnormally upregulated Mitophagy and Xenophagy.

Below we summarize the unanswered questions in the field of selective autophagy hoping for greater attention and *ad hoc* investigation:

1. How many common traits between mitophagy and xenophagy remain unaddressed?
2. Which are the consequences on cellular pathophysiology of the shared amplificatory role of TBK1 in mitophagy and xenophagy when both these pathways are activated?
3. Could the high degree of conservation between bacterial and mammalian genomes of the antimitophagy protein TSPO represent an exploitable target to maximize the therapeutic value of xenophagy to co-adjuvate antimicrobial therapy?

## Author Contributions

All authors listed have made a substantial, direct and intellectual contribution to the work, and approved it for publication.

## Conflict of Interest Statement

The authors declare that the research was conducted in the absence of any commercial or financial relationships that could be construed as a potential conflict of interest.
